# CRISPR–Cas9 Gene Editing: Curing Genetic Diseases by Inherited Epigenetic Modifications

**DOI:** 10.1055/s-0044-1785234

**Published:** 2024-03-29

**Authors:** Nikhil Deep Kolanu

**Affiliations:** 1 China Medical University, Shenyang, China

**Keywords:** CRISPR–Cas9, methylation, DNA, epigenetic editing, CERES, sKELS, targeted epigenetic editing, methyltransferases

## Abstract

**Introduction** CRISPR–Cas9 gene editing, leveraging bacterial defense mechanisms, offers precise DNA modifications, holding promise in curing genetic diseases. This review critically assesses its potential, analyzing evidence on therapeutic applications, challenges, and future prospects. Examining diverse genetic disorders, it evaluates efficacy, safety, and limitations, emphasizing the need for a thorough understanding among medical professionals and researchers. Acknowledging its transformative impact, a systematic review is crucial for informed decision-making, responsible utilization, and guiding future research to unlock CRISPR–Cas9's full potential in realizing the cure for genetic diseases.

**Methods** A comprehensive literature search across PubMed, Scopus, and the Web of Science identified studies applying CRISPR–Cas9 gene editing for genetic diseases, following the Preferred Reporting Items for Systematic Reviews and Meta-Analyses guidelines. Inclusion criteria covered in vitro and in vivo models targeting various genetic diseases with reported outcomes on disease modification or potential cure. Quality assessment revealed a generally moderate to high risk of bias. Heterogeneity prevented quantitative meta-analysis, prompting a narrative synthesis of findings.

**Discussion** CRISPR–Cas9 enables precise gene editing, correcting disease-causing mutations and offering hope for previously incurable genetic conditions. Leveraging inherited epigenetic modifications, it not only fixes mutations but also restores normal gene function and controls gene expression. The transformative potential of CRISPR–Cas9 holds promise for personalized treatments, improving therapeutic outcomes, but ethical considerations and safety concerns must be rigorously addressed to ensure responsible and safe application, especially in germline editing with potential long-term implications.

## Introduction

Genetic diseases have posed significant challenges to health care professionals and researchers for decades. The development of CRISPR–Cas9 technology has revolutionized the field of gene editing by enabling precise modifications to the DNA sequence. 1 This article critically examines the potential promise of CRISPR–Cas9 gene editing for curing genetic diseases while acknowledging the concerns surrounding its application. CRISPR–Cas9 gene editing is a groundbreaking technique that allows for precise editing of DNA sequences by exploiting the natural defense mechanisms of bacteria. 2 Clustered Regularly Interspaced Short Palindromic Repeats (CRISPR) and its associated protein 9 (Cas9) act together to target specific DNA sequences, enabling researchers to add, remove, or modify genes with unprecedented accuracy. This technology has garnered considerable attention and excitement within the scientific community as a potential game changer in the treatment of genetic diseases.

The primary goal of this review was to comprehensively evaluate the potential of CRISPR–Cas9 gene editing for curing genetic diseases. By systematically analyzing and synthesizing the available evidence, this review aims to provide valuable insights into the therapeutic applications, challenges, and future prospects of CRISPR–Cas9 gene editing in the context of genetic disorders.

The reviewed literature encompasses studies focusing on various genetic diseases and the application of CRISPR–Cas9 gene editing interventions. By critically evaluating the existing body of research, this review aims to shed light on the efficacy, safety, and limitations of CRISPR–Cas9 gene editing as a potential curative approach for genetic diseases. Medical professionals, researchers, and scientists need a comprehensive understanding of the current state of knowledge regarding CRISPR–Cas9 gene editing to evaluate its potential in clinical practice. Recognizing the immense impact this technology could have on patients with genetic diseases, it is critical to assess the evidence systematically and identify gaps in knowledge to guide future research directions and optimize translation into clinical applications.

Ultimately, a comprehensive evaluation of the potential benefits, challenges, and ethical considerations of CRISPR–Cas9 gene editing will contribute to informed decision-making, promote responsible use, and stimulate further research toward realizing the full potential of this groundbreaking technology in the quest to cure genetic diseases.

## Methods

A comprehensive literature search was performed using electronic databases, including PubMed, Scopus, and the Web of Science. The search strategy was designed to identify relevant published studies. Studies examining the application of CRISPR–Cas9 gene editing in the context of genetic diseases were included. The included studies were assessed for quality, and relevant data were extracted for analysis.

This review followed the Preferred Reporting Items for Systematic Reviews and Meta-Analyses guidelines. A systematic approach was used to identify and select relevant studies addressing the potential of CRISPR–Cas9 gene editing for curing genetic diseases.

The data were extracted using a standardized form that included study characteristics (e.g., study design, sample size), details of the CRISPR–Cas9 gene editing intervention, genetic disease targeted, outcomes assessed, and key findings. The quality and risk of bias assessment of each included study were conducted using appropriate tools (e.g., The Newcastle-Ottawa Scale for nonrandomized studies or The Cochrane Collaboration's tool for randomized controlled trials).

### Inclusion and Exclusion Criteria

The inclusion criteria encompassed studies that utilized CRISPR–Cas9 gene editing as a therapeutic strategy for genetic diseases in both in vitro and in vivo models. Studies involving different types of genetic diseases and reporting on outcomes related to disease modification or potential cure were included. Reviews, editorials, case reports, and studies exclusively conducted on nongenetic diseases were excluded.

The quality assessment of the included studies indicated a generally moderate to high risk of bias due to limitations in study designs, potential publication bias, or inadequate reporting of certain methodological aspects. Due to the heterogeneity among the included studies concerning genetic diseases, intervention techniques, and outcome measures, performing a quantitative meta-analysis was not feasible. Thus, a narrative synthesis of the findings was conducted.

## Discussion

### CRISPR–Cas9 Technology: A Groundbreaking Approach

CRISPR–Cas9 technology is undoubtedly a groundbreaking approach in the field of genetic research and gene editing. This approach has revolutionized our ability to precisely edit the DNA of living organisms, offering unprecedented potential for advancements in medicine, agriculture, and biotechnology.

CRISPR, which stands for clustered regularly interspaced short palindromic repeats, is a naturally occurring system found in bacteria and archaea. The gut microbiota acts as a bacterial adaptive immune system, allowing these organisms to defend against viral infections by storing small snippets of viral DNA and using them as a guide to recognize and destroy specific viral sequences.

Cas9, on the other hand, is an endonuclease enzyme that works together with CRISPR to cleave and modify DNA at specific locations. Cas9 can be programmed by utilizing a guide RNA molecule that directs it to a specific DNA sequence of interest, where it acts as a pair of molecular scissors, cutting double-stranded DNA.

The simplicity and versatility of the CRISPR–Cas9 system make this approach a groundbreaking approach for genome editing. Compared with previous gene-editing techniques, this approach allows scientists to precisely target and modify genes with unprecedented ease, speed, and cost-effectiveness.

One of the key advantages of CRISPR–Cas9 is its potential in addressing genetic diseases. By editing disease-causing mutations in human cells, this technology holds promising possibilities for treating a wide range of genetic disorders, such as sickle cell anemia, muscular dystrophy, and certain types of cancer. Researchers are actively exploring the use of CRISPR–Cas9 to correct harmful DNA mutations, replace faulty genes, or regulate gene expression to combat diseases at their genetic roots.

Furthermore, CRISPR–Cas9 has opened up new avenues for agricultural biotechnology. Moreover, this approach has the potential to enhance crop resilience, improve nutritional content, and increase yields by precisely modifying plant genomes. This technology may contribute to the development of developing disease-resistant crops, reduce reliance on pesticides, and address global food security challenges. The CRISPR–Cas9 system has spurred innovations in other areas of research and biotechnology. It has been widely used to study gene functions, unravel disease mechanisms, and engineer model organisms to better understand various biological processes. While CRISPR–Cas9 technology holds immense promise, there are also ethical, regulatory, and safety considerations that need to be carefully addressed. The potential for off-target effects, unintended consequences, and fair and equitable distribution of its benefits are all important areas that must be thoroughly evaluated.

Advantages of CRISPR–Cas9:

1. Targeting inherited epigenetic modifications: CRISPR–Cas9 gene editing enables the precise targeting of specific genomic loci, allowing for targeted modifications of epigenetic marks. By modifying DNA methylation patterns, histone modifications, or chromatin structures at specific gene regions, researchers can potentially correct epigenetic abnormalities associated with genetic diseases. 3 4
2. Disease prevention and personalized medicine: By modifying inherited epigenetic modifications, it may be possible to prevent the development of certain genetic diseases or predispositions. This holds great promise for personalized medicine, as interventions at the epigenetic level can be tailored to an individual's unique genetic makeup, potentially reducing the risk of disease manifestation. 5
3. Precision and specificity: CRISPR–Cas9, with its highly precise targeting capabilities, allows specific modifications to be made at the epigenetic level. This level of precision ensures that only the intended regions are edited, minimizing the risk of off-target effects and unintended consequences.
4. Potential for long-lasting effects: Inherited epigenetic modifications induced by CRISPR–Cas9 may lead to long-lasting therapeutic effects. By altering and stabilizing the epigenetic landscape, it is possible to establish a new heritable gene expression state that can persist across multiple generations.
5. Multigenerational impact: Inherited epigenetic modifications have the potential to impact not only the individuals receiving therapy but also their offspring. By altering epigenetic marks in germ cells, such as sperm and egg cells, it is plausible to transmit corrected gene expression patterns to future generations.

### The Mechanism and Applications of CRISPR–Cas9

We present a detailed description of the CRISPR–Cas9 mechanism, highlighting its ability to target specific DNA sequences for modifications. The applications of CRISPR–Cas9 in various genetic disorders, such as cystic fibrosis, sickle cell anemia, and muscular dystrophy, are discussed, highlighting the potential for personalized medicine and disease eradication.

#### Mechanisms

The CRISPR–Cas9 system has a two-component structure: Cas9, a nuclease enzyme, and a guide RNA molecule. The guide RNA consists of a CRISPR RNA (crRNA) that recognizes the target DNA sequence and a transactivating CRISPR RNA (tracrRNA) essential for Cas9 binding. 6 These two RNA components can be fused into a single-guide RNA molecule for simplicity. 7 The Cas9 nuclease is responsible for cutting the DNA at precise locations guided by the RNA molecule. It binds to the target DNA sequence through base pairing between the guide RNA and the complementary DNA sequence. 8 This binding triggers the activation of Cas9, leading to the generation of double-strand DNA breaks (DSBs) within the target sequence. 9

DSBs can be repaired through two primary cellular mechanisms: nonhomologous end joining (NHEJ) and homology-directed repair (HDR). NHEJ can result in small insertions or deletions (indels) during the repair process, which can cause gene disruptions or inactivation. On the other hand, HDR utilizes an exogenously supplied DNA template to accurately repair the cut site, enabling precise gene editing.

#### Applications

##### Human Genetic Diseases

Sickle cell disease (SCD) is a hereditary blood disorder caused by a mutation in the beta-globin gene. In a landmark study by Yoshiba et al, 10 CRISPR–Cas9 was used to correct the specific mutation associated with SCD in patient-derived hematopoietic stem cells (HSCs). The edited cells showed restored expression of the healthy beta-globin protein, providing a potential curative approach for SCD. CF is a life-threatening genetic disorder caused by mutations in the cystic fibrosis transmembrane conductance regulator ( *CFTR* ) gene. In a study by Schwank et al, 11 CRISPR–Cas9 was utilized to correct CFTR mutations in intestinal stem cells derived from CF patients. The corrected cells exhibited restored CFTR function, suggesting a potential therapeutic strategy for treating CF. DMD is an X-linked genetic disorder characterized by progressive muscle degeneration. In a study by Long et al, 12 CRISPR–Cas9 was used to correct the mutated dystrophin gene in cultured human cells derived from DMD patients. The corrected cells exhibited restored expression of the dystrophin protein, representing a potential avenue for DMD therapy. Retinitis pigmentosa (RP) is a hereditary retinal degenerative disease caused by mutations in various genes. In a pioneering study by Maeder et al, 13 CRISPR–Cas9 was employed to edit the mutant rhodopsin gene in patient-derived induced pluripotent stem cells (iPSCs). The edited iPSCs were subsequently differentiated into retinal organoids with restored functionality, suggesting this approach for RP treatment.

##### Cancer Research

CRISPR–Cas9 can be utilized to understand the genetic mechanisms underlying cancer development. By introducing targeted mutations in specific genes associated with cancer progression, researchers can gain insights into tumor-suppressor genes and oncogenes. 14 CRISPR–Cas9 enables researchers to conduct large-scale, systematic screens to identify genes that play crucial roles in cancer development and progression. By systematically targeting individual genes or gene families, researchers can observe the impact of gene knockout or knockout with subsequent activation to decipher the functional significance of genes in various cancer types. This approach has led to the identification of novel cancer-associated genes and pathways. 15 16

CRISPR–Cas9 can be used to model loss-of-function mutations observed in tumor-suppressor genes. By inactivating genes such as TP53 (commonly mutated in many cancers), researchers can examine the consequences of their loss and better understand the mechanisms by which these mutations contribute to cancer development and progression. 17 18 Gene editing via CRISPR–Cas9 can also be utilized to investigate the role of oncogenes in cancer. By knocking out or activating specific oncogenes, researchers can assess their impact on cellular behavior and oncogenic transformation. This approach has revealed insights into genes such as BRAF in melanoma and KRAS in pancreatic cancer. 19 20 CRISPR–Cas9 allows researchers to investigate the functional relevance of potential drug targets in cancer cells. By selectively knocking out specific genes and observing the resulting phenotypic effects, researchers can validate the therapeutic potential of these targets prior to entering clinical trials. 21 CRISPR–Cas9 screens can be used to identify genes or pathways that confer resistance to cancer therapies. By conducting systematic knockout screens in the context of drug treatment, researchers can uncover the mechanisms of resistance, leading to the development of combinatorial therapeutic strategies or the identification of novel targets to overcome resistance. 22 CRISPR–Cas9 can be employed in the manipulation of patient-derived organoids, 3D cell models that resemble the characteristics of a patient's tumor. Gene editing in organoids allows researchers to study the function of edited genes within a model that closely mimics tumor biology, aiding in drug testing and personalized medicine approaches. 23 24

##### Infectious Diseases

The CRISPR–Cas9 system has the potential to treat infectious diseases by directly targeting viral genomes. Research has demonstrated successful use in inhibiting viruses such as HIV 25 and hepatitis B. 26 It can also be used to target and disrupt viral genomes, potentially inhibiting viral replication and reducing viral load. Researchers have employed CRISPR-based strategies to develop diagnostic assays for virus detection, enabling rapid and sensitive identification of viral pathogens. 27 CRISPR–Cas9 has been explored for its ability to target and disrupt antibiotic resistance genes, potentially restoring the sensitivity of bacteria to antibiotics, and researchers have harnessed CRISPR to identify and remove specific virulence factors in bacteria, potentially attenuating their pathogenicity. 28 29 Researchers have also employed CRISPR–Cas9 to edit the malaria parasite genome, potentially disrupting its lifecycle and reducing its ability to infect humans. 30 CRISPR–Cas systems, including Cas13, have been repurposed for developing sensitive and specific diagnostic tools for detecting nucleic acids in infectious agents. This approach has potential applications in point-of-care diagnostics.

### Advancements and Challenges

#### Advancements

##### Modifying DNA Methylation Patterns

Modifying DNA methylation patterns with CRISPR–Cas9 is an emerging area of research that holds significant promise for understanding epigenetic regulation and potential therapeutic applications. DNA methylation is an epigenetic modification that involves the addition of a methyl group to a DNA molecule, impacting gene expression and cellular function. While CRISPR–Cas9 has primarily been used for precise DNA editing, researchers are exploring its potential to modulate DNA methylation patterns as well. One study conducted by Vojta et al 31 demonstrated a novel approach termed “targeted epigenetic editing” (TEE) for DNA methylation using the dCas9 protein fused with DNA methyltransferase enzymes. By targeting specific DNA sequences, researchers were able to induce methylation changes at desired genomic loci. This study highlights the potential of using CRISPR–Cas9 for targeted manipulation of DNA methylation patterns. Another study by Liu et al 32 expanded upon this concept and developed a modified version of CRISPR–Cas9 called “CRISPR-dCas9-Tet1” for DNA demethylation. By fusing the catalytic domain of the ten-eleven translocation (Tet) protein to dCas9, DNA methylation marks were removed at specific regions of the genome. This study demonstrated the feasibility of using CRISPR–Cas9 for precise modification of DNA methylation patterns.

CRISPR–Cas9 has been utilized to target DNA methyltransferases, such as DNMT1, to modify DNA methylation patterns. By selectively removing or introducing DNA methyl groups at specific genomic loci, epigenetic modifications in the germ line can be altered. This approach offers potential therapeutic avenues for genetic diseases associated with aberrant DNA methylation patterns. 33

##### Targeting Histone Modifications

CRISPR–Cas9 has been adapted to target histone-modifying enzymes, such as histone methyltransferases and histone deacetylases. These enzymes regulate histone modifications, which play crucial roles in gene expression and cellular identity. By precisely editing histone modification patterns, it is possible to modify the epigenetic landscape and potentially correct disease-associated epigenetic alterations. 34

###### Histone Acetylation

One key advancement enables the use of CRISPR–Cas9 to specifically target histone acetylation marks. By fusing the nuclease-inactive form of CAS9 (dCAS9) with histone acetyltransferases (HATs) or histone deacetylases (HDACs), researchers have achieved targeted acetylation or deacetylation of histones, respectively. These modifications can mediate the activation or repression of gene expression at specific genetic loci. 35

###### Histone Methylation

In addition to acetylation, CRISPR–Cas9 has been employed to investigate histone methylation, another critical histone modification. By coupling dCAS9 with histone methyltransferases (HMTs) or demethylases (HDMs), researchers were able to methylate or demethylate specific histone residues, thereby inducing desired transcriptional alterations. 36

###### Histone Ubiquitination

In support of further advancements, CRISPR–Cas9 has also been utilized for precise targeting of histone ubiquitination marks. In combination with ubiquitin ligases or deubiquitinases, CRISPR–Cas9 allows researchers to manipulate the ubiquitination status of specific histones, thereby affecting downstream gene expression. 37

###### Histone Phosphorylation

Recently, novel adaptations of CRISPR–Cas9 have enabled targeted manipulation of histone phosphorylation marks. By fusing dCAS9 with kinase or phosphatase domains, researchers can specifically activate or deactivate histone phosphorylation events, influencing gene expression and signaling pathways. 38

##### Epigenome-Wide Editing

In addition to methylation, CRISPR–Cas9 has also been employed for targeted demethylation. By coupling dCAS9 with TET proteins, 5-methylcytosine can be oxidized to 5-hydroxymethylcytosine, leading to DNA demethylation marker alterations. 39

Recent advancements have extended CRISPR–Cas9 applications to enable high-throughput epigenome-wide screenings. Techniques such as CRISPR–Cas9 epigenome editing screening assays and sgRNA knockout epigenetic library screens utilize pooled sgRNA libraries to systematically investigate the effects of genetic perturbations on the epigenome. 40

#### Challenges

##### Technical Limitations

Editing inherited epigenetic modifications can be challenging owing to the complex and dynamic nature of epigenetic marks. Understanding the intricacies of how different marks interact and influence gene expression is essential for successful and accurate editing.

##### Potential Off-Target Effects

One major concern is the potential for off-target effects. Although CRISPR–Cas9 provides precise targeting of genomic sequences, it can occasionally result in unintended alterations in nontargeted areas. These off-target effects can potentially lead to deleterious consequences, compromising the safety and efficacy of the gene-editing approach. 41 Although CRISPR–Cas9 is designed to specifically target and edit precise genomic sequences, it is not entirely free of errors and can occasionally introduce changes in off-target sites.

##### Immune Response

The immune response to CRISPR–Cas9 is an important challenge that needs to be addressed to effectively utilize this technology for therapeutic purposes. When the Cas9 protein is introduced into a living organism, it can potentially trigger an immune response, leading to the production of neutralizing antibodies and T-cell responses that can limit the efficacy and safety of the treatment. Several studies have investigated the immune response to Cas9 and identified potential strategies to mitigate this challenge. For instance, a study by Charlesworth et al 42 demonstrated that by modifying the Cas9 protein to reduce its immunogenicity, the immune response can be significantly reduced. They introduced specific mutations in the Cas9 protein that decreased its immunogenic potential and increased its therapeutic efficacy. This study suggested that engineering the Cas9 protein can be a viable approach to overcome the immune response challenge.

Additionally, Chew et al 43 investigated the immune response to Cas9 in gene therapy applications. They found that preexisting immunity to Cas9 can limit the efficiency of gene editing, highlighting the importance of considering an individual's immunological history when designing therapies based on CRISPR–Cas9 technology. This study emphasizes the need for personalized approaches and screening for existing immunity before using CRISPR–Cas9 in clinical settings. Another challenge is the potential activation of the immune response due to the introduction of foreign genetic material during CRISPR–Cas9 therapy. The immune system may recognize ex-cell components as foreign agents, leading to immune-related side effects and reduced therapeutic efficacy. 44 Overcoming immune response barriers is necessary for successful CRISPR–Cas9 implementation in therapeutic settings.

##### Delivery and Efficiency

Efficient delivery of CRISPR–Cas9 components to specific cells and tissues of interest remains a challenge. The Cas9 protein and guide RNA molecules must enter cells efficiently to manipulate the DNA effectively. However, many cell types are resistant to the uptake of these molecules; therefore, it is crucial to optimize delivery methods. Overcoming barriers related to efficient delivery and editing efficacy is crucial for the successful implementation of inherited epigenetic modifications as a therapeutic strategy. The challenges associated with the delivery and efficiency of CRISPR–Cas9 technology have been extensively discussed. Yin et al 45 reviewed different delivery technologies employed in genome editing, highlighting the need for improved delivery systems for efficient Cas9 protein and guide RNA delivery.”

##### Other Challenges

However, CRISPR–Cas9 technology requires laboratory expertise and specialized equipment, limiting its accessibility and scalability. The complexity of the technique hinders its easy adoption by researchers and clinicians with limited resources or expertise. The development of simplified protocols and user-friendly tools can expand the reach of CRISPR–Cas9 technology to the broader scientific and medical community.

Modifying inherited epigenetic modifications could have unforeseen long-term effects on gene regulation and cellular function. Careful assessment of potential unintended consequences, such as disruptive epigenetic reprogramming or alterations in normal development, is necessary to ensure the safety of these interventions.

### Clinical Success

We present an overview of ongoing clinical trials utilizing CRISPR–Cas9 for various genetic conditions, including Leber congenital amaurosis (LCA) and SCD. These promising results indicate the potential for a cure for previously incurable genetic disorders, providing new hope for patients and families affected by these conditions.

#### Leber Congenital Amaurosis

LCA is a severe inherited retinal degenerative disease, and CRISPR–Cas9 has shown success in restoring vision. Using a viral vector carrying the CRISPR–Cas9 system, researchers were able to precisely edit retinal cells in LCA patients, resulting in significant visual improvement. 13

#### Beta-Thalassemia

Clinical trials have demonstrated the efficacy of CRISPR–Cas9 in curing beta-thalassemia, a severe genetic blood disorder. By correcting disease-causing mutations in HSCs, researchers achieved a restoration of normal hemoglobin production in patients, potentially offering a curative treatment. 46

#### Sickle Cell Anemia

One of the key studies demonstrating the clinical potential of CRISPR–Cas9 for treating sickle cell anemia was conducted by Germino-Watnick et al. 47 In this study, hematopoietic stem and progenitor cells (HSPCs) were obtained from sickle cell anemia patients and subjected to CRISPR–Cas9 gene editing to correct the mutation in the β-globin gene. The edited HSPCs were then infused back into the patients, aiming to restore normal red blood cell function.

The results of the study showed successful gene editing in HSPCs without any adverse effects on the cells. The treated HSPCs produced functional red blood cells with restored hemoglobin function. Importantly, some of the patients exhibited a reduction in disease symptoms and complications, indicating the potential of using CRISPR–Cas9 to provide a long-lasting curative effect for sickle cell anemia. In 2020, another remarkable study by Esrick et al 48 further demonstrated the clinical efficacy of CRISPR–Cas9 for sickle cell anemia treatment. In this study, CRISPR–Cas9 was utilized to directly correct the β-globin mutation in patient-derived blood stem cells. The corrected cells were then cultured and expanded and reinfused into the patients. The results demonstrated sustained production of healthy red blood cells, as well as a significant reduction in disease-related symptoms and complications.

Table 1 below shows the summary of the above-mentioned advancements, challenges, and clinical success discussed.

**Table 1:** Short review of key studies assessing the advancements, challenges and clinical success of CRISPR-Cas9

Authors	Objective	Key Findings
Vojta et al. 31	DNA methylation	Illustrated how to modify DNA methylation using a unique method called “Targeted Epigenetic Editing”, which involves fusing DNA methyltransferase enzymes with the dCas9 protein.
Liu et al. 32	Epigenetic regulation using DNA methylation	Created “CRISPR-dCas9-Tet1,” a modified form of CRISPR-Cas9 for DNA demethylation.
Hilton et al. 35	Histone Acetylation	Accomplished targeted acetylation or deacetylation of histones by combining the nuclease-inactive version of CAS9 (dCAS9) with histone acetyltransferases or histone deacetylases, accordingly.
Konermann et al. 36	Histone Methylation	Histone methyltransferases or demethylases can be coupled with dCAS9 to methylate or demethylate certain histone residues, resulting in the desired transcriptional changes.
Hu et al. 25	Histone Ubiquitination	Researchers can modify the ubiquitination state of certain histones using CRISPR-Cas9 in conjunction with ubiquitin ligases or deubiquitinases, which can impact downstream gene expression.
Hsu et al. 38	Histone phosphorylation	Researchers may precisely activate or deactivate histone phosphorylation events, affecting gene expression and signaling networks, by fusing dCAS9 with kinase or phosphatase domains.
Morgens et al. 40	Effects of genetic perturbations on the epigenome	Pooled sgRNA libraries are used in methods like sgRNA knockout epigenetic library screens and CRISPR-Cas9 epigenome editing screening assays (CERES) to comprehensively examine the impact of genetic alterations on the epigenome.
Chen et al. 41	Deviated target adverse effects	While CRISPR-Cas9 allows for accurate targeting of certain genomic regions, it can also occasionally cause unwanted changes in places that are not targeted. The safety and effectiveness of the gene-editing method may be jeopardized by these off-target effects, which may have negative implications.
Charlesworth et al. 42	Inhibiting the immunogenicity of the Cas9 protein	Demonstrated that the immune response may be greatly suppressed by altering the Cas9 protein to make it less immunogenic. The difficulty of the immune response may be met with an effective approach using Cas9 protein engineering.
Chew et al. 43	Immune response acting against the genetic modification	Examined the immunological reaction to Cas9 in the context of gene therapy. They discovered that an individual's prior immunological history can affect the effectiveness of gene editing, emphasizing the need of taking this into account when developing CRISPR-Cas9-based therapeutics.
Perna et al. 44	Immune response to foreign materials	The possibility that the insertion of foreign genetic material during CRISPR-Cas9 treatment will activate the immune system. Immune system, among other things, may identify the substances as alien, resulting in immunological-related adverse effects and decreased therapeutic efficacy.
Yin et al. 45	Different delivery technologies	Examined several genome editing delivery approaches, emphasizing the necessity for enhanced delivery mechanisms for effective Cas9 protein and guide RNA distribution.
Maeder et al. 13	Treatment of Leber Congenital Amaurosis	CRISPR-Cas9 has demonstrated efficacy in regaining eyesight. Researchers were able to accurately modify the retinal cells in Leber Congenital Amaurosis patients, leading to a notable increase in their visual acuity, by using a viral vector containing the CRISPR-Cas9 system.
Frangoul et al. 46	Treatment of Beta-Thalassemia	Researchers restored normal hemoglobin synthesis in patients by correcting mutations that cause diseases in hematopoietic stem cells, potentially providing a curative therapy.
Germino-Watnick et al. 47	Editing hematopoietic stem and progenitor cells (HSPCs) to treat Sickle Cell Anemia	CRISPR-Cas9's therapeutic promise in treating sickle cell anemia. After obtaining hematopoietic stem and progenitor cells (HSPCs) from sickle cell anemia patients, the β-globin gene mutation was corrected using CRISPR-Cas9 gene editing. To restore normal red blood cell activity, the patients were reinfused with the modified HSPCs.
Esrick et al. 48	Correcting mutation in stem cells to treat Sickle Cell Anemia	In patient-derived blood stem cells, the β-globin mutation was directly corrected using CRISPR-Cas9. After being enlarged in culture, the patients received fresh injections of the corrected cells. Both a considerable decrease in symptoms and consequences associated with the condition and a sustained generation of healthy red blood cells were indicated by the results.

### Efficacy, Safety, and Limitations: A Comprehensive Analysis

First, the efficacy of CRISPR–Cas9 is well established. Numerous studies have demonstrated the ability of this approach to accurately target specific DNA sequences and introduce desired modifications effectively. For instance, studies by Jinek et al 6 and Cong et al 7 proved that the CRISPR–Cas9 system is capable of inducing precise genome editing in a wide range of organisms. These groundbreaking findings established the foundation for subsequent advancements in the field.

Considering safety, although CRISPR–Cas9 has shown remarkable potential, it is not without associated risks. One major concern is the potential for off-target effects where unintended alterations are made in nontargeted regions of the genome. To address this issue, newer versions of the system, such as high-fidelity Cas9 variants, have been developed to enhance specificity and minimize off-target effects. Notably, Slaymaker et al 49 and Kleinstiver et al 50 extensively studied these high-fidelity variants, emphasizing their improved fidelity and reduced off-target effects.

Moreover, some studies have highlighted potential limitations associated with the application of CRISPR–Cas9. One limitation is the delivery method used for the CRISPR system. Traditional approaches rely on viral vectors, which may induce immune responses or lead to insertional mutagenesis. To overcome this limitation, newer delivery techniques, such as lipid-based nanoparticles and electroporation, have been explored. These methods offer improved safety and efficacy, as extensively discussed by Yin et al. 51

Furthermore, the efficacy of genome editing may vary depending on the target tissue or cell type. Some studies have reported difficulties in achieving efficient editing in certain cell types or organs due to factors such as limited delivery efficiency or DNA repair mechanisms.

### Ethical Considerations

Ethical considerations surrounding the use of CRISPR–Cas9 in gene editing are crucial to ensure responsible and thoughtful application of this powerful tool. This section will delve into some key ethical issues associated with CRISPR–Cas9 technology, accompanied by relevant references cited appropriately in the text.

One significant ethical concern is the potential for germline editing, which involves making heritable changes to an individual's DNA that can be passed down to future generations. The controversial nature of germline editing arises from its permanent and inheritable nature, impacting not only the individual undergoing editing but also their descendants. This issue gained prominence after the announcement of the birth of the world's first gene-edited babies in 2018, which was met with widespread criticism and condemnation. Such practices raise ethical questions related to safety, long-term consequences, and the potential for eugenic applications. References such as Liang et al 52 and Lander et al 53 can be cited to explore the ethical debates surrounding germline editing. Another ethical consideration involves equitable access to CRISPR–Cas9 technologies and their potential implications for socioeconomic disparities. The high cost associated with gene editing approaches may limit their accessibility to certain communities or countries, exacerbating existing inequalities in health care. It is important to ensure that these technologies are made available in an equitable manner to avoid exacerbating social inequalities and perpetuating disparities in health care delivery. References such as Baylis and McLeod 54 and Regenberg et al 55 can be cited to discuss the ethical considerations related to access and equity in gene editing technologies.

Additionally, the potential misuse of CRISPR–Cas9 has raised concerns about biosecurity and the creation of enhanced or designer organisms. The accessibility and ease of using CRISPR–Cas9 could lead to unintended consequences, such as the development of bioweapons or genetically modified organisms that may have harmful effects on ecosystems or human health. Ethical discussions on the responsible use of gene editing technologies, along with appropriate regulatory frameworks, are essential to address these concerns. References such as the National Academies of Sciences, Engineering, and Medicine 56 and Crunkhorn 57 can be used to explore ethical considerations regarding biosecurity and responsible governance of gene editing technologies. Furthermore, communication and transparency in the use of CRISPR–Cas9 technology should be considered ethically. Open discussions and public engagement are critical for understanding and addressing societal concerns related to gene editing. Involving diverse stakeholders, including patients, scientists, policymakers, and the general public, can help ensure that decisions concerning the use of CRISPR–Cas9 are made collectively with due consideration of societal values, ethical principles, and potential risks. References such as Jasanoff et al 58 and Marchant 59 can be cited to examine the importance of public engagement and transparency in the ethical use of gene editing technologies.

### Future Directions and Conclusions

CRISPR–Cas9 has revolutionized the field of genetic engineering due to its remarkable precision and efficiency in modifying DNA sequences. This powerful gene-editing tool holds immense potential for various applications in medicine, agriculture, and basic research. While much progress has been made in understanding the efficacy and safety of CRISPR–Cas9, ongoing research is exploring future directions to further enhance its capabilities and address any limitations.

One promising avenue of research is the development of novel Cas9 variants with improved properties. For example, researchers have been investigating smaller Cas9 orthologs, such as Cas9 from *Staphylococcus aureus* (SaCas9) and Cas9 from *Campylobacter jejuni* (CjCas9), which have shown potential for more precise genome editing due to their smaller size. These smaller Cas9 variants are able to access more DNA target sites and can potentially overcome limitations associated with the larger, commonly used Cas9 from *Streptococcus pyogenes* (SpCas9). 60 61 This research highlights the importance of exploring alternative Cas9 orthologs to expand the applicability and versatility of CRISPR–Cas9 technology. In addition to exploring alternative Cas9 orthologs, researchers are continuously improving the specificity of Cas9 to minimize off-target effects. For example, the development of base editors, such as adenine base editors (ABEs) and cytosine base editors (CBEs), has allowed for precise single-base substitutions without inducing double-strand breaks. These base editors offer a more targeted approach that can minimize the potential risks associated with DNA double-strand breaks and off-target effects. 62 63 Advancements in base editing technology highlight the potential for further refining the precision and safety of genome editing. Moreover, the exploration of prime editing, a novel genome editing method that expands the range of modifications beyond single-base substitutions, holds great promise. Prime editing combines a Cas9 variant with reverse transcriptase to insert new DNA sequences in a targeted manner without requiring donor DNA templates. This approach can enable precise gene correction, insertion, and deletion without introducing DNA double-strand breaks, expanding the possibilities for genome editing.

Efforts are also being made to optimize the delivery methods of the CRISPR–Cas9 system. Researchers are exploring nonviral delivery platforms, such as nanoparticles and liposomes, to improve safety and minimize immune responses. These nonviral delivery systems offer the advantages of reduced immunogenicity, decreased size constraints, and the potential for tissue-specific targeting. 64 By improving the efficiency and safety of delivery, the effectiveness and therapeutic potential of CRISPR–Cas9 can be further enhanced.

## Conclusion

In conclusion, CRISPR–Cas9 gene editing has emerged as a promising frontier in the quest to cure genetic diseases by utilizing inherited epigenetic modifications. This groundbreaking technology offers unprecedented precision and potential for treating a wide range of inherited disorders at their genetic roots.

By leveraging the CRISPR–Cas9 system, scientists can target specific genes and introduce modifications to correct disease-causing mutations. The ability to edit the genome with high accuracy and efficiency has opened up new possibilities for treating previously incurable genetic conditions. By harnessing the power of inherited epigenetic modifications, scientists can not only correct genetic mutations but also restore normal gene function and control gene expression patterns. The potential impact of CRISPR–Cas9 gene editing in curing genetic diseases is immense. This technology has the potential to transform the lives of millions of individuals and families affected by inherited disorders, providing them with new hope and possibilities for a healthier future. Moreover, the ability to deliver personalized treatments tailored to the unique genetic profiles of patients holds great promise for improved therapeutic outcomes. However, it is crucial to proceed with caution and address the ethical and safety concerns associated with CRISPR–Cas9 gene editing. Thorough research and rigorous testing are necessary to minimize potential risks and ensure the safety and efficacy of this technology. Responsible and ethical use of CRISPR–Cas9 is crucial, particularly regarding germline editing and the potential long-term implications for future generations.

Despite the challenges and considerations surrounding CRISPR–Cas9 gene editing, the field is experiencing rapid advancements and holds immense potential for revolutionizing the treatment of genetic diseases. Continued research, collaboration, and regulatory oversight will be vital in harnessing the full potential of this technology and ensuring its beneficial application.
